# H_2_-Producing Bacterial Community during Rice Straw Decomposition in Paddy Field Soil: Estimation by an Analysis of [FeFe]-Hydrogenase Gene Transcripts

**DOI:** 10.1264/jsme2.ME16036

**Published:** 2016-06-18

**Authors:** Ryuko Baba, Susumu Asakawa, Takeshi Watanabe

**Affiliations:** 1 Laboratory of Soil Biology and Chemistry, Department of Biological Mechanisms and Functions, Graduate School of Bioagricultural Sciences, Nagoya UniversityFurocho, Chikusa, Nagoya 464–8601Japan

**Keywords:** paddy field soil, [FeFe]-hydrogenase, hydrogen-producing microorganism, anoxic soil incubation, clone library analysis

## Abstract

The transcription patterns of [FeFe]-hydrogenase genes (*hydA*), which encode the enzymes responsible for H_2_ production, were investigated during rice straw decomposition in paddy soil using molecular biological techniques. Paddy soil amended with and without rice straw was incubated under anoxic conditions. RNA was extracted from the soil, and three clone libraries of *hydA* were constructed using RNAs obtained from samples in the initial phase of rice straw decomposition (day 1 with rice straw), methanogenic phase of rice straw decomposition (day 14 with rice straw), and under a non-amended condition (day 14 without rice straw). *hydA* genes related to *Proteobacteria*, *Firmicutes*, *Bacteroidetes*, *Chloroflexi*, and *Thermotogae* were mainly transcribed in paddy soil samples; however, their proportions markedly differed among the libraries. *Deltaproteobacteria-*related *hydA* genes were predominantly transcribed on day 1 with rice straw, while various types of *hydA* genes related to several phyla were transcribed on day 14 with rice straw. Although the diversity of transcribed *hydA* was significantly higher in the library on day 14 with rice straw than the other two libraries, the composition of *hydA* transcripts in the library was similar to that in the library on day 14 without rice straw. These results indicate that the composition of active H_2_ producers and/or H_2_ metabolic patterns dynamically change during rice straw decomposition in paddy soil.

Paddy fields are flooded during most of the rice cultivation period, leading to anoxic conditions in bulk soil, except for the thin surface layer of soil and a part of rhizosphere soil. Nitrate, Mn (IV, III), Fe (III), and sulfate are sequentially reduced depending on the redox potentials of the substances, and methanogenesis is initiated under the most reduced conditions (13, 32). Rice straw, left on the field after rice harvesting, is commonly incorporated into soil and one of the main sources of soil organic matter. Rice straw, which contains easily decomposable organic substances, is the main origin of methane emitted from paddy fields (39). The decomposition of rice straw in soil is processed with the transient accumulation of some intermediates such as sugars, volatile fatty acids, and alcohols (7, 10, 20, 35). Hydrogen (H_2_) is one of the key intermediates produced during rice straw decomposition; however, the apparent production of H_2_ is low in paddy field soil (12). H_2_ production is the sink of excess reducing equivalents, which are supplied to H_2_ consumers, and regulates anaerobic decomposition processes (5, 12). The microorganisms responsible for rice straw decomposition have been investigated in several studies (11, 24, 28, 30, 36, 40). However, the diversity and dynamics of H_2_ producers as a functional group related to rice straw decomposition have not yet been elucidated.

Hydrogenases are enzymes that catalyze H_2_ production and consumption, and have been grouped into three classes: [NiFe]-, [FeFe]-, and [Fe]-hydrogenases. [FeFe]-hydrogenases are distributed in the *Bacteria* and *Eukarya* domains. These enzymes are the main H_2_-producing enzymes in anaerobic environments (3, 23, 37); however, certain [NiFe]-hydrogenases catalyze the production of H_2_ from formate (37). [FeFe]-hydrogenases exist as monomeric or polymeric FeS proteins, and contain a region called the H cluster, which is encoded by *hydA* (37). *hydA* genes harbor conserved regions (37) that enable the design of specific primers to analyze H_2_-producing bacterial communities. Although the phylogenetic resolution of *hydA* gene sequences is lower than that of 16S rRNA genes, mainly due to gene duplication and lateral gene transfer (23, 26, 37), the phylogenetic analysis of *hydA* in environments is important for linking the production of H_2_ with the diversity and dynamics of H_2_-producing bacterial communities. *hydA* genes have recently been used as a marker gene to elucidate the diversity of H_2_-producing bacteria in environments such as ethanol-H_2_ co-producing systems (41), anoxic sewage sludge (34), acidic fen soil (26), microbial mats (2), termite gut (42) and earthworm gut contents (27). In addition, since the transcriptional levels of *hydA* have been correlated to H_2_ production rates in some *Clostridium* species (18, 38), active members of H_2_ producers may be evaluated using an RNA-based analysis.

We previously investigated the diversity of H_2_-producing bacteria in paddy field soil using a clone library analysis (1). Predominant members of potential H_2_-producing bacteria were composed of a wide range of groups including *Proteobacteria*, *Firmicutes*, *Bacteroidetes*, and *Chloroflexi*. However, the active members of H_2_ producers in paddy field soil have not yet been identified. Since the soil redox potential and decomposition pathway of rice straw vary dynamically after flooding in paddy field soil, the composition of key H_2_ producers in soil is assumed to shift with soil reduction and rice straw decomposition processes.

Therefore, we herein attempted to identify the active members of H_2_-producing bacteria and their compositional changes during rice straw decomposition in anoxic paddy field soil. In soil incubation experiments, a molecular biological analysis targeting *hydA* for soil DNAs and RNAs was performed in order to elucidate transcriptional patterns under the different decomposition processes of rice straw in paddy soil.

## Materials and Methods

### Soil and rice straw samples

Soil samples were collected from a paddy field located at the Aichi-ken Anjo Research and Extension Center, central Japan (Anjo field; latitude 34°58′21″N, longitude 137°04′35″E) on 2 October 2013. The chemical properties of Anjo soil were as follows: total C, 14.5 g kg^−1^; total N, 1.4 g kg^−1^; pH [H_2_O], 5.2; free iron content, 5.96 g kg^−1^. This soil is classified as Oxyaquic Dystrudepts (29) with a Light Clay texture. An approximately 1-kg composite plowed layer soil sample (0–10 cm) was collected into a plastic bag from four randomly selected spots in the field using a trowel. Soil samples were then passed through a 2-mm mesh sieve, mixed thoroughly, and stored at 4°C until used. Rice straw (*Oryza sativa* L. Aichinokaori SBL, obtained from the Anjo field) was pulverized using Wonder Blender WB-1 (Osaka Chemical, Osaka, Japan), and passed through a 0.5-mm mesh sieve.

### Incubation of soil

Ten grams of soil with and without 0.05 g of powdered rice straw was added to 4 mL of distilled water in a screw cap test tube (φ18 × 180 mm; Sanshin Industrial, Yokohama, Japan) and mixed well. The tube was closed with a butyl rubber stopper and screw cap (Sanshin Industrial). The treatments containing soil with and without rice straw were designated as treatment R and treatment N, respectively. The gas phase in the tubes was replaced with N_2_ using a Deoxygenized Gas Pressure & Replace Injector (MODEL IP-8, Sanshin Industrial). The tubes were incubated at 25°C in the dark without shaking, and soil samples were collected 0, 1, 3, 7, 14, 20, and 28 days after the beginning of the incubation. Triplicate tubes were prepared for each sampling time, except for the measurement of gas production (*n*=6, the same tubes were subjected to measurements throughout the experiment).

### Measurement of gases, nitrate, ferrous iron, and sulfate

Gas samples (1 mL) were collected with a Pressure-Lok^®^ glass syringe (Valco Instruments Company, Baton Rouge, LA, USA) from the gas phase in closed test tubes after vortex-mixing, and the concentrations of carbon dioxide (CO_2_) and methane (CH_4_) were measured using the gas chromatograph GC-14B equipped with TCD and FID (Shimadzu, Kyoto, Japan), respectively, and Porapak N^®^ (80–100 mesh; GL Sciences, Tokyo, Japan). H_2_ was measured using GC-7A equipped with TCD (Shimadzu) and Molecular sieve 5A (60–80 mesh). After the measurement of these gases, the gas phase was again replaced with N_2_.

Ferrous iron in incubated soil (1 g) was extracted using acetate buffer (pH 3.0), and the concentrations of ferrous iron in samples were measured by the colorimetric method using *o*-phenanthroline (31).

Sulfate and nitrate were extracted from the collected soil (2 g) by shaking for one hour with 10 mL distilled water. The suspensions were filtered with No. 6 Quantitative Filter Paper (Advantec, Tokyo, Japan) and 0.20-μm syringe filters (Advantec). The filtrates were stored at 4°C until used for measurements. The concentrations of anions were measured using an ion chromatograph PIA-1000 (Shimadzu) equipped with a Shima-pack IC-A3(S) (Shimadzu) by a conductivity detection method. The mobile phase containing 8 mM *p*-hydroxybenzoic acid and 3.2 mM Bis-Tris was flowed at a rate of 0.20 mL min^−1^ at 35°C (column temperature).

### Nucleic acid extraction

Regarding nucleic acid extraction, 0.5 g (wet weight) of soil was collected in a 2-mL microtube (Sarstedt, Nümbrecht, Germany) with 0.7 g of zirconium beads (diameter, 0.1 mm), frozen by liquid N_2_, and stored at −80°C. Total nucleic acids were extracted from soil, and DNA was digested with DNase I (Promega, Madison, WI, USA) according to the procedure described by Murase *et al.* (19). RNA dissolved in RNase-free TE buffer was stored at −80°C. The complete digestion of DNA in RNA samples was confirmed by PCR using the bacterial universal primer set 357f-GC/517r (21) in the absence of reverse transcriptase. Nucleic acid mixtures without the DNase treatment were used as DNA samples for the clone library analysis on day 0 and a DGGE analysis (see Supplemental document). cDNA was synthesized from each RNA sample using the PrimeScript^®^ RT reagent Kit (Perfect Real Time) (Takara, Otsu, Japan) with a random 6-mer according to the manufacturer’s instructions.

### Clone library analysis of *hydA* and transcripts

PCR targeting partial *hydA* sequences (*ca.* 600 bp: 610–703 bp) was performed for cDNA and DNA samples using the primer set HydH1f/HydH3r (26) with the modified PCR program (1). In order to amplify *hydA*, 5 μL of cDNA and 1.8 μL of a 10-fold dilution of extracted DNA were used for each 45-μL or 28.2-μL reaction premix. Procedures for the purification of PCR products, construction of clone libraries for the DNA sample on 0 day with rice straw (day0), cDNA samples on 1 and 14 days with rice straw (day1R and day14R) and 14 days without rice straw (day14N), and cycle sequencing were described previously (1). Plasmid DNA was extracted using NucleoSpin^®^ Plasmid EasyPure (Macherey-Nagel, Düren, Germany). A sequencing analysis was performed with an Applied Biosystems 3500 Genetic Analyzer (Applied Biosystems, Foster City, CA, USA).

Sixty clones from each sample were analyzed and approximately 50 potential *hydA* partial sequences were obtained. Chimera sequences were checked by UCHIME *de novo* (6). The nucleotide sequences of the clones were translated to amino acid sequences (189–220 aa) using the EMBOSS Transeq program (EMBL-EBI [http://www.ebi.ac.uk/Tools/st/emboss_transeq/]). Amino acid sequences containing the L2 signature (PCxxKxxE) (17, 22, 37) were used for further analyses. The closest relatives of *in silico*-translated *hydA* sequences were searched using the BLAST program on the NCBI website (http://blast.ncbi.nlm.nih.gov/) between March and June 2014 as described by Baba *et al.* (1). Comparisons of libraries were performed by a weighted UniFrac analysis (15) using Mothur 1.32.1 (25) with the neighbor-joining tree constructed by ClustalW 1.83 on the DDBJ website (version 1.83; DDBJ [http://clustalw.ddbj.nig.ac.jp/]) with default parameters. The criteria of operational taxonomic units (OTUs) and diversity indices (Ace, Chao1 and Shannon) were determined in the threshold of 80% sequence similarity by Mothur according to previous studies (1, 27). A principal component analysis based on the OTU distribution of each sample obtained by Mothur was performed using R (version 3.1.1; R Foundation for Statistical Computing [http://www.R-project.org/]). Data on the OTU distribution were standardized before the analysis because the number of sequences of each library was different. The Tukey-Kramer test for diversity indices, and a clustering analysis by Ward’s method for UniFrac significances were performed using the R. A phylogenetic tree was constructed with representative amino acid sequences deduced from transcribed *hydA* and reference sequences by the neighbor-joining method with ClustalW 2.1 on the DDBJ website under the default parameters, and the tree was formatted using MEGA 5.2 (33). [FeFe]-hydrogenase-like Narf protein sequences (Accession no. P23503, Q6CGR3, and Q8SYS7) were used as the outgroup sequences.

### Accession numbers

The nucleotide sequences of *hydA* determined in the clone libraries have been deposited to the DDBJ database under accession numbers LC041370–LC041941.

## Results

### Sequential reduction processes of paddy soil

**Fig. 1:**
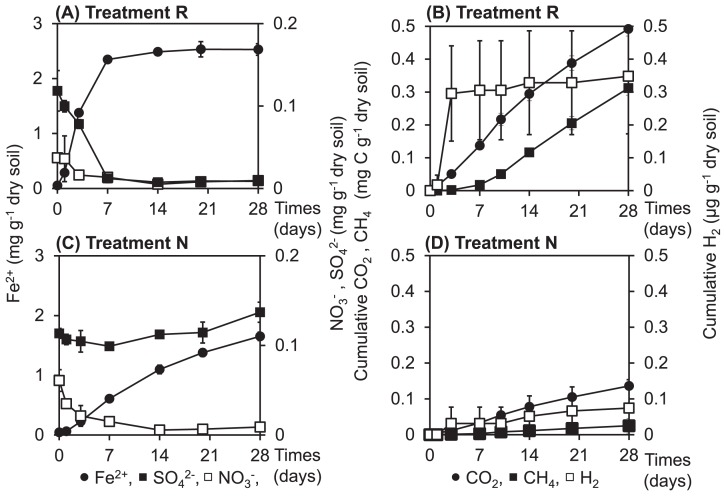
Changes in NO_3_^−^, Fe^2+^, and SO_4_^2−^ concentrations (A, C) and the production of CO_2_, H_2_, and CH_4_ (B, D) in treatment R (A, B) and treatment N (C, D).

In treatment R (the treatment of soil incubated with rice straw), the concentration of nitrate decreased until day 14, while that of ferrous iron rapidly increased and reached a maximum (*ca.* 2.5 mg g^−1^ dry soil) within one week (Fig. 1A). In addition, the concentration of sulfate markedly decreased during the initial 7 days in treatment R (Fig. 1A). Apparent H_2_ production was only observed between 1 day and 3 day. CO_2_ was actively produced immediately after day 1, while active CH_4_ production occurred after day 7 in treatment R (Fig. 1B).

In treatment N (the treatment of soil incubated without rice straw), the concentration of nitrate decreased until day 14, while that of ferrous iron linearly increased until day 28 (*ca.* 1.5 mg g^−1^ dry soil, Fig. 1C). However, in contrast to treatment R, the concentration of sulfate remained unchanged in treatment N during the incubation (Fig. 1C). Changes in the production of H_2_, CH_4_, and CO_2_ were similar between treatments N and R; however, the amounts of gases produced were markedly smaller in treatment N (Fig. 1D).

### Distinct members transcribing *hydA* in paddy soil under different soil conditions

Considering the soil conditions described above, four clone libraries (*hydA*, DNA library of day 0 in treatment R [day0]; *hydA* transcripts, cDNA libraries of day 1 and day 14 in treatment R [day1R and day14R, respectively], and day 14 in treatment N [day14N]) were constructed to show the dominant members that transcribed *hydA* in soil during rice straw decomposition.

**Fig. 2:**
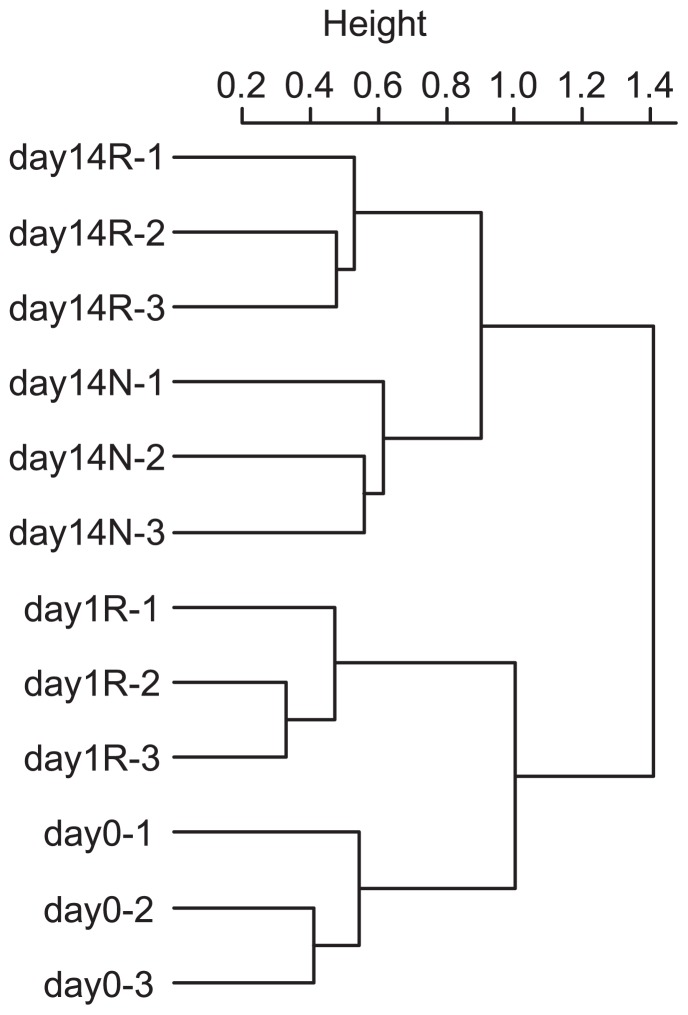
Cluster dendrogram representing similarities and differences among libraries. The value of weighted UniFrac significances was used for the calculation. The number after the dash of each sample represents its replication number. day0, DNA library of day 0 in treatment R (incubation with rice straw); day1R, cDNA libraries of day 1 in treatment R; day14R, cDNA libraries of day 14 in treatment R; day14N, cDNA libraries of day 14 in treatment N (incubation without rice straw).

**Fig. 3:**
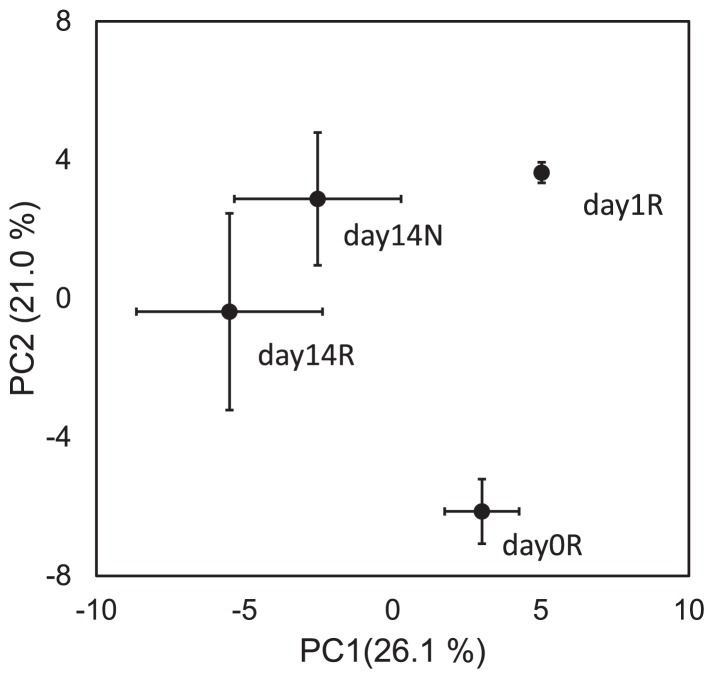
Principal component analysis of the OTU distribution in day0, day1R, day14N, and day14R (*n*=3). PC1 and PC2 mean the scores of the first and second principal components. The percentages in parentheses mean the contribution rates of PC1 and PC2. The error bars show the standard deviation of each library.

The number of OTUs and diversity indices of Chao1, Ace, and Shannon are shown in Table 1. All diversity indices were significantly higher on day14R than on day1R and day14N (Table 1; *P*<0.05); however, no significant difference was observed between day0 and other libraries (day1R, day14N, and day14R) or between day1R and day14N. The clustering analysis based on the value of weighted UniFrac significances showed that the replicates of each library were clustered into the same groups depending on the type of the treatment and incubation day (Fig. 2). The principal component analysis based on the distribution of OTUs in the libraries also showed similar results to those of the clustering analysis (Fig. 3).

**Fig. 4:**
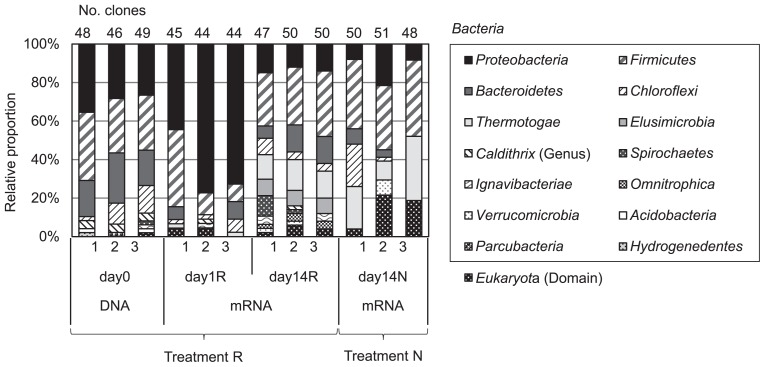
Phylogenetic composition of clones in *hydA* libraries. The numbers below the bar graph indicate replications.

The proportion of closest microbial phyla obtained by a BLAST analysis in each library is shown in Fig. 4. The ranges of the identities and similarities of the amino acid sequences deduced from *hydA* in this study to the known [FeFe]-hydrogenase partial sequences were 44–97% and 58–98%, respectively. The lowest and highest similarities were obtained from the amino acid sequences derived from *Chlamydomonas reinhardtii* (EDP02498) and *Clostridium saccharoperbutylacetonicum* N1-4(HMT) (AGF57068), respectively. Various phyla were observed in each library. The sequences of *Bacteroidetes*-, *Chloroflexi*-, *Firmicutes*-, and *Proteobacteria*-related *hydA* transcripts were detected in all libraries, while the sequences of *Thermotogae*-related *hydA* transcripts were only observed in cDNA libraries (day1R, day14N, and day14R). The sequences of *Eukaryota*-related *hydA* transcripts were also observed in (some or all replicates of) all libraries, although the number was small. Sixty-five and twenty percent of the sequences in day1R were related to the [FeFe]-hydrogenases of *Proteobacteria* and *Firmicutes*, respectively. *Desulfovibrio*- and *Pelobacter*-related *hydA* transcripts (the similarity ranges of deduced [FeFe]-hydrogenase sequences were 77–93% and 90–94%, respectively) occupied more than 40% of all clones obtained in day1R. On the other hand, in day14R, *Proteobacteria*-related *hydA* transcripts were markedly decreased, while the proportions of *Firmicutes*-, *Bacteroidetes*-, and *Thermotogae*-related *hydA* transcripts slightly increased. In day14N, more than 30% of the sequences were close to *Firmicutes*-related [FeFe]-hydrogenases, similar to day14R. The proportion of *Thermotogae*-related *hydA* transcripts was also higher than that in day1R.

**Fig. 5:**
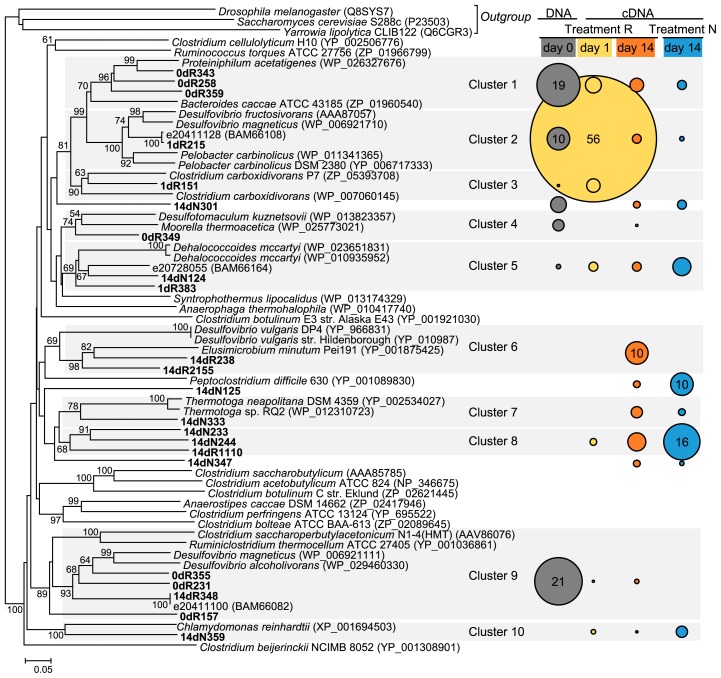
Phylogenetic tree of deduced amino acid sequences of *hydA* in representative OTUs containing more than five clones, and the proportion of clones in subclusters to libraries. Clones with bold names indicate amino acid sequences derived from *hydA* transcripts obtained in this study. The numbers at the branches indicate bootstrap values (≥50%) with 1,000 replicates. Clustering (shown in grey squares) is based on phylogenetic relationships among the sequences. The sizes of circles and numbers on the right column show the relative abundance and percentages (≥10%) of clones belonging to each cluster among all clones in each library.

A phylogenetic tree of known [FeFe]-hydrogenase partial sequences and the representative sequences obtained in this study, which were derived from the major OTUs containing more than 5 clones, is shown in Fig. 5. Most of the OTUs were grouped into 10 clusters according to their phylogenetic relationships. The phylogenetic distributions and proportions of [FeFe]-hydrogenases were different among the libraries, as shown in Fig. 2, 3, and 4. The proportion of Cluster 2 in Fig. 5, which was related to the [FeFe]-hydrogenases of *Desulfovibrio* and *Pelobacter*, was markedly different among the libraries; it was 56% in day1R, but equal to or less than 10% in the other libraries. Some of the representative OTUs, *e.g.*, the OTUs in Clusters 6, 7, 8, and 10, were only observed in the cDNA libraries (day1R, day14N, and day14R).

## Discussion

H_2_ metabolism in anoxic paddy field soil affects the reduction processes and decomposition pathway of organic matter in soil. The present study analyzed transcripts of *hydA*, which encode possible H_2_-producing enzymes [FeFe]-hydrogenases, under different soil conditions (day1R, the ferric iron and sulfate reduction phase, and initial phase of rice straw decomposition; day14R, the phase of methanogenesis and mid-phase of rice straw decomposition; day14N, the phase of ferric iron reduction and substrate-poor conditions) in order to identify active members of H_2_ producers during rice straw decomposition in paddy field soil.

*hydA* libraries constructed from DNA samples in this study suggested that members of *Proteobacteria*, *Firmicutes*, *Bacteroidetes*, and *Chloroflexi* were the main H_2_ producers existing in paddy soil (Fig. 4). A DGGE analysis of *hydA* showed that the band patterns did not change during the incubation period (Fig. S1A and B in the Supplemental document). Our previous study, investigating *hydA* diversity in a double cropping paddy field, also showed the predominance of *Proteobacteria*, *Firmicutes*, *Bacteroidetes*, and *Chloroflexi* and no significant differences in the community between drained and flooded soil (1). Thus, these results suggest that the community structure of H_2_ producers is stable during rice straw decomposition in paddy field soil. However, the distribution of transcribed *hydA* (day1R, day14N, and day14R) was clearly distinguished from those of *hydA* (day0) based on the UniFrac-based clustering analysis and OTU-based principal component analysis (Fig. 2 and 3). Although the diversity of *hydA* does not always indicate the diversity of H_2_ producers because of multiple *hydA* genes in some bacteria, the results shown in Fig. 2 and 3 imply that the active community structure of H_2_ producers in paddy field soil differed from existing H_2_ producers and/or the H_2_ metabolic patterns (of H_2_ producers) changed.

The clone library analysis showed that the transcriptional patterns of *hydA* genes in paddy soil were markedly different among the three libraries (Fig. 2 and 3). In day1R, when the initial decomposition of rice straw and rapid ferric iron and sulfate reduction occurred (Fig. 1), the proportion of Cluster 2, which was close to *Deltaproteobacteria*-related [FeFe]-hydrogenases, was high (Fig. 4 and 5). In day14R, under methanogenic conditions with the active decomposition of rice straw, various H_2_ producers transcribed *hydA* (Table 1, Fig. 4 and 5). On the other hand, in day14N, when ferric iron reduction occurred, but sulfate reduction did not under relatively substrate-poor conditions, the proportions of the other types of [FeFe]-hydrogenases were relatively high (Clusters 6, 7, and 8 in Fig. 5). These results suggest that various active H_2_ producers participate in the decomposition of rice straw and/or soil reduction processes, and there were dynamic changes in the transcriptional activity of *hydA* in paddy field soil under different soil conditions.

In day1R, the initial phase of rice straw decomposition, the emission of the excess amount of H_2_ produced and the active reduction of iron and sulfate were observed. This kind of phase was characterized by the high activity levels of the fermentation of reducing sugars and relatively low activity levels of H_2_ consumption (8). Therefore, H_2_ producers fermenting reducing sugars may have become active in day1R. Weber *et al.* showed that specific members of *Clostridia* dominated the microbial community at an early phase of shredded rice straw decomposition using PCR-DGGE analyses (40). In the present study, we also showed the lowest diversity of transcribed *hydA* in day1R with the dominance of *Deltaproteobacteria*, which was also indicated to be responsible for the decomposition of rice straw incorporated into paddy field soil (30) and the decomposition of rice straw on rice roots (28). Itoh *et al.* showed that the proportion of some *Deltaproteobacteria*-related 16S rRNA was higher in flooded paddy soil than in drained soil (9). They suggested that some *Deltaproteobacteria* play important roles in sulfate reduction and H_2_ production under anoxic conditions in paddy field soil. Although further studies are needed in order to elucidate the role of *hydA* transcribed in this phase in more detail, the results of the present study indicated that microorganisms possessing *Deltaproteobacteria*-related *hydA* were responsible for a part of H_2_ production in the initial phase of rice straw decomposition and soil reduction.

In day14R, ferric iron and sulfate reduction were almost completed, and methanogenesis occurred (Fig. 1). In the methanogenic phase, reducing sugars accumulated by the hydrolysis of rice straw polysaccharides were generally almost consumed (8), and intermediate metabolites such as VFAs may be the main substrates for fermentation (8, 10, 35). Therefore, typical secondary fermenters such as *Syntrophobacter* (16) and *Syntrophomonadaceae*-related bacteria (14) were expected to produce H_2_ by transcribing *hydA* during the oxidization of VFAs. However, our results suggest that a phylogenetically broader range of fermenters transcribed *hydA* and produced H_2_ in day14R (Table 1, Fig. 4 and 5) than in day1R and day14N. This result is consistent with previous findings showing that various microorganisms (24, 30, 40) were involved in rice straw decomposition. In addition, a distinct pattern of transcription of *hydA* was observed in day14R, but overlapped with that in day14N to some extent (Fig. 2 and 3). Therefore, various types of *hydA* appeared to have been transcribed in the methanogenic phase of rice straw decomposition. This result indicates that a large number of H_2_ producers are responsible for rice straw decomposition and produce H_2_; however, the diversity of *hydA* does not necessarily indicate the diversity of H_2_ producers because of multiple *hydA* in their genome (17, 22). Methanogenesis from H_2_ and CO_2_ mostly occur via interspecies electron transfer (4), and ATP synthesis by H_2_-producing primary fermenters increases when they grow with hydrogenotrophic methanogens (43). Therefore, not only specific secondary fermenters, but also various fermenters may have grown with methanogens by interspecies H_2_ transfer in the methanogenic phase of rice straw decomposition.

When soil was relatively organic substrate-poor in the non-methanogenic early phase (in day14N), the phylogenetic distribution of transcribed *hydA* was different from that in day1R (Fig. 2, 4 and 5). On the other hand, some *hydA* genes were commonly transcribed in day14N and day14R (Fig. 5), and a similar transcriptional pattern was observed to some extent (Fig. 2, 3 and 4). Glissmann and Conrad showed that the same types of volatile fatty acids accumulated in a similar manner in the incubation of rice-straw amended and non-amended paddy soil (7). Therefore, the types of active H_2_ producers in rice-straw amended and non-amended paddy soil may be common to some extent. However, soil conditions (organic matter content and soil redox conditions) were markedly different between day14R and day14N (Fig. 1). OTU-based diversities were higher in day 14R (Table 1). Therefore, although some *hydA* transcripts were common to two libraries, the active community structures of H_2_ producers appeared to differ between day14R and day14N.

In conclusion, we herein revealed the dynamics of potentially active members of H_2_ producers during rice straw decomposition in paddy soil using molecular biological techniques targeting *hydA* transcripts. Our results showed that specific *hydA* genes were transcribed in the initial phase of rice straw decomposition and soil reducing processes. More diversified *hydA* genes were transcribed, *i.e.,* more various H_2_ producers may have become active in the methanogenic phase than in the non-methanogenic phase. Although the role and function of these microorganisms remain to be elucidated and further studies are required, the results obtained herein suggest that *hydA* transcriptional patterns and/or active members of H_2_ producers in paddy field soil are dynamically changed in the process of rice straw decomposition and soil redox conditions.

## Supplementary Information



## Figures and Tables

**Table 1:** Number of sequences, OTUs, and diversity indices of *hydA* libraries obtained in this study.

		Treatment	Library number	No. Clones	No. OTU^a^	Coverage^b^	Ace		Chao1		Shannon	
DNA	day 0	R	1	48	19	0.79	30	**AB**	42	**AB**	2.7	**AB**
			2	46	29	0.52	132		145		3.2	
			3	49	28	0.67	46		48		3.2	

mRNA	day 1	R	1	45	17	0.80	30	**A**	23	**A**	2.3	**A**
			2	44	11	0.80	56		29		1.1	
			3	44	15	0.80	28		21		1.8	

	day 14	R	1	47	31	0.57	57	**B**	55	**B**	3.3	**B**
			2	50	35	0.56	63		54		3.5	
			3	50	34	0.50	90		84		3.4	

		N	1	50	18	0.84	37	**A**	23	**A**	2.5	**A**
			2	51	19	0.82	36		31		2.7	
			3	48	21	0.85	25		25		2.9	

a The criterion of OTUs was assessed in the threshold of 80% sequence similarity, according to previous studies (1, 27).

b The criterion of coverages was C=1-n_1_/N, in which n_1_ is the number of OTUs that have been sampled once and N is the total number of sequences in each sample.

**A** and **B** indicate the results of the Tukey-Kramer test: data with different letters show a significant difference (*P*<0.05).
