# Supplementary material for: H2-Producing Bacterial Community during Rice Straw Decomposition in Paddy Field Soil: Estimation by an Analysis of [FeFe]-Hydrogenase Gene Transcripts
Source: Microbes Environ. 2016 Jun 18;31(3):226–33. doi: 10.1264/jsme2.ME16036 (PMC5017798; doi:10.1264/jsme2.ME16036)
Supplement: Supplementary file 1 [file 31_226_s1.pdf]

Supplemental document

H_2-producing bacterial community during the rice straw decomposition in a paddy field soil: estimation by the analysis of transcripts of [FeFe]-hydrogenase gene

Ryuko Baba, Susumu Asakawa, Takeshi Watanabe

Materials and methods

To show the dynamics of existing and active members of H_2 producers during the incubation, DGGE analysis was performed using DNA and cDNA (mRNA) samples. A PCR targeting *hydA* sequences was performed for the DNA and cDNA samples using the primer set HydH1f-33GC/HydH3r (2)(GC clamp was designed in Throbäck *et al.* (3)) with the modified PCR program (1). The PCR products were purified after gel extraction of targeting bands (ca. 650 bp) from agarose gels as described in Baba *et al.* (1). Purified PCR products (ca. 100-150 ng) were subjected to DGGE analyses with denaturant gradient range 30-70% and photographed as described previously (4). However, because small amount of PCR products were obtained from cDNA samples in the treatment N when HydH1f-33GC was used, therefore we did not perform the DGGE analysis for these samples.

Results and discussion

The PCR-DGGE analysis showed that the *hydA* banding patterns obtained from the DNA samples did not change during the incubation in the both treatment N and R (Fig. S1A and B). The results indicated that the community structure of H_2 producers was stable under the different soil conditions.

In contrast, the DGGE banding patterns of *hydA* originated from the mRNA samples of the treatment R were changed during the incubation (Fig. S1C). The results

indicated the transcription activity of *hydA* in the community was changed during the incubation. Although faint banding patterns were observed in some samples, especially in day14 of the mRNA samples (Fig. S1C), this might be because various types of bacteria transcribed *hydA*.

References

1. Baba, R., M. Kimura, S. Asakawa, and T. Watanabe. 2014. Analysis of [FeFe]-hydrogenase genes for the elucidation of a hydrogen-producing bacterial community in paddy field soil. FEMS Microbiol. Lett. 350:249-256.
2. Schmidt, O., H.L. Drake, and M.A. Horn. 2010. Hitherto unknown [Fe-Fe]-hydrogenase gene diversity in anaerobes and anoxic enrichments from a moderately acidic fen. Appl. Environ. Microbiol. 76:2027-2031.
3. Throbäck, I.N., K. Enwall, Å. Jarvis, and S. Hallin. 2004. Reassessing PCR primers targeting *nirS*, *nirK* and *nosZ* genes for community surveys of denitrifying bacteria with DGGE. FEMS Microbiol. Ecol. 49:401-417.
4. Watanabe, T., S. Asakawa, A. Nakamura, K. Nagaoka, and M. Kimura. 2004. DGGE method for analyzing 16S rDNA of methanogenic archaeal community in paddy field soil. FEMS Microbiol. Lett. 232:153-163.

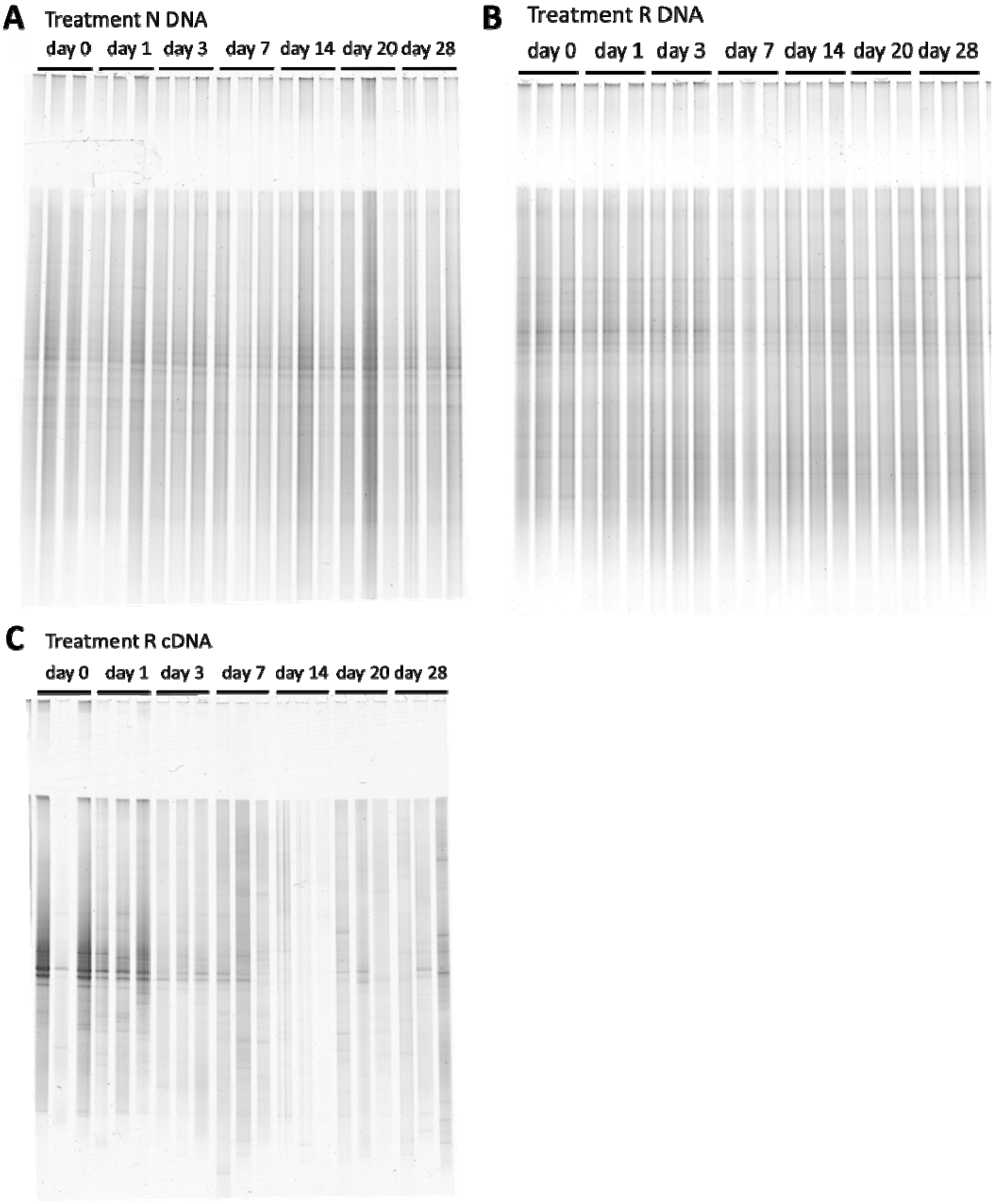

Fig. S1 DGGE banding patters of *hydA* PCR products during the incubation in (A, B) DNA and (C) cDNA samples of both (B) treatment N and (B, C) R. Three lanes of each incubation period indicate the samples obtained from the triplicate incubations.
